# Size Matters: Large Objects Capture Attention in Visual Search

**DOI:** 10.1371/journal.pone.0015293

**Published:** 2010-12-23

**Authors:** Michael J. Proulx

**Affiliations:** Biological and Experimental Psychology Group, School of Biological and Chemical Sciences, Queen Mary University of London, London, United Kingdom; Center for Genomic Regulation, Spain

## Abstract

Can objects or events ever capture one's attention in a purely stimulus-driven manner? A recent review of the literature set out the criteria required to find stimulus-driven attentional capture independent of goal-directed influences, and concluded that no published study has satisfied that criteria. Here visual search experiments assessed whether an irrelevantly large object can capture attention. Capture of attention by this static visual feature was found. The results suggest that a large object can indeed capture attention in a stimulus-driven manner and independent of displaywide features of the task that might encourage a goal-directed bias for large items. It is concluded that these results are either consistent with the stimulus-driven criteria published previously or alternatively consistent with a flexible, goal-directed mechanism of saliency detection.

## Introduction

A crucial survival tool for an organism is the ability to orient toward important aspects of the environment and to ignore irrelevant distractions. One beneficial result of this orienting is the efficient selection of those aspects of the environment that are consistent with one's goals. Humans are particularly dependent on the visual modality for analyzing the environment. Within vision alone, at any given moment there is light entering the eye from all angles and providing us with a visual field full of more information than we can interpret. Attention is the mechanism by which features, objects, and spatial locations in the environment are selected for increased scrutiny, thus allowing an organism to selectively extract from the environment the information that is most needed to achieve current goals.

An interaction of the goal-directed behavior of the organism and the stimulus-driven nature of the environment determines the speed and accuracy with which one can visually search for an object [1]. The relative influence of these processes on the deployment of attention has been under much scrutiny for the past two decades. The focus of many studies has been whether features or events can capture attention in a primarily stimulus-driven manner, and evidence for and against this abound (for a review, see [2]).

In a recent review of the attentional capture literature, a number of criteria were proposed to assess whether evidence of attentional capture was due to stimulus-driven or goal-directed processes [3]. These criteria were derived from research demonstrating that the demands and design of a task (‘displaywide features’, see [4]) often result in goal-directed cognitive control settings that include the attention-capturing feature or event.

How can such goal-directed processes be ruled out? Burnham [3] proposed the following requirements for the attention-capturing feature or event: (a) it must be irrelevant to the target-defining features of the task as instructed by the experimenter; and (b) it must be irrelevant to any learned strategies adopted by the participant. The key criterion is the latter, as any incidental aspect of the task might induce the participant to attend to a non-target-defining feature. For example, the objects to be searched through commonly appear on the screen by an abrupt visual onset on a blank background (see, e.g., [5]). Even if the participant is searching for a particular letter, the informativeness of the dynamic onset of the items to initialize the search task might result in an attentional control setting for not only the letter target, but for dynamic events as well. Thus if the abrupt appearance of a new item captured attention, it might be due to goal-directed processes that prioritize dynamic events [4].

Experiment 1 provided the first published test of whether a large item can capture attention when one is engaged in efficient search for orientation. Size was manipulated in terms of object length such that the large item would not necessarily appear as closer in depth. Recently Zehetleitner et al. [6] provided the first evidence that a singleton (a bright time) could capture attention in a visual search task requiring simple, efficient target detection. This result was surprising because previous studies had only demonstrated such capture in compound tasks where one must localize a target based on one set of criteria (such as object shape) and respond based on another set (the orientation of a line enclosed by the shape) [7]. This led some to propose a dual-route hypothesis for visual search [8], in contrast to single-route models such as Guided Search [9]. Note that the Zehetleitner et al. [6] study used the common ‘additional singleton’ paradigm where the attention-capturing bright item was never at the target location (see also [7]).

The present experiment extends this result by using the irrelevant feature paradigm, where the unique, task-irrelevant large feature appeared at the target location with chance probability, consistent with Burnham's first criterion. Note also that because the length singleton was present on every trial, it is possible that it serves as a display-onset signal or an implicitly learned feature of the display; however, it is more likely that the participants would learn an attentional set for a dynamic onset rather than for length in predicting the start of each trial. Indeed, with only one item as the larger item on each trial, amongst a variety of display lengths, there are many more features beside the length singleton that would more likely be used as an attentional set arising from a display onset signal that do not favor length in particular. This first experiment is thus crucial to establish whether the long singleton is salient enough to capture attention in a detection task, using the irrelevant feature paradigm, and in an efficient search task.

Because Experiment 1 presented an efficient search task for an orientation singleton, the resulting attentional capture by a singleton in another dimension (length) is expected due to goal-directed processes. Consistent with the idea that an observer can learn an alternative strategy, it was expected that the option to detect a salient singleton [10], rather than attend to the target-defining feature of orientation, would result in attentional capture by a singleton, such as the large but irrelevant feature that appeared in the display.

Experiment 2 provided a test of the attention-capturing ability of large objects with Burnham's [3] criteria in mind. The second experiment tested whether the large item would capture attention in a task not expected to promote a learned strategy to detect a salient singleton [10]. The task was made inefficient by increasing target-nontarget similarity for the orientation detection task (from 90 degrees orientation contrast in Experiment 1 to 30 degrees orientation contrast in Experiment 2). If the large item was prioritized in this experiment, then this would provide evidence for stimulus-driven attentional capture by a static, large singleton that was neither a target-defining feature nor a feature that indicated the search should commence, as with the dynamic onset of all the items.

## Results

### Experiment 1: Efficient Search

**Figure 1:**
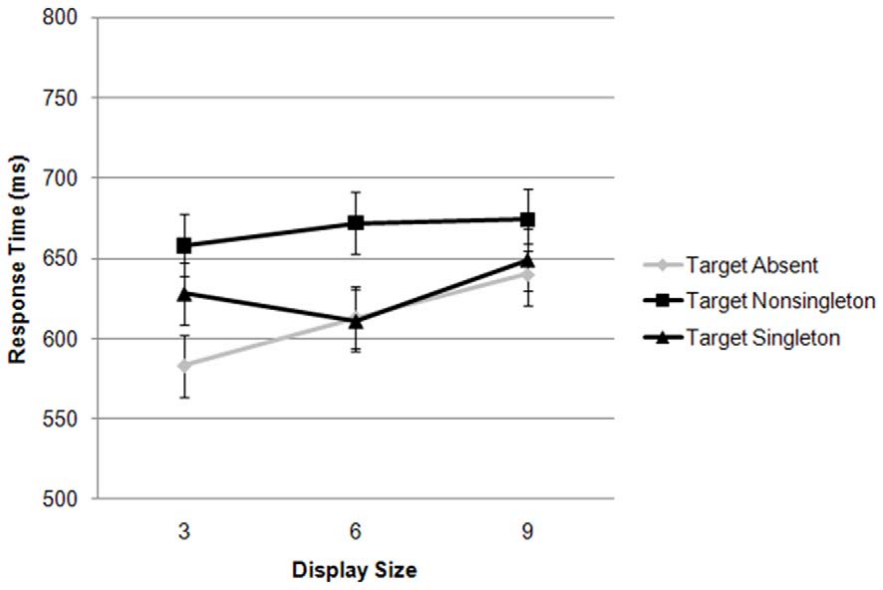
The response times for visual search for a right-tilted target bar among left-tilted nontargets (see **Figure 4** panels A and B) as a function of display size for the target absent, target-present nonsingleton, and target-present singleton conditions are plotted. Error bars in this and all RT plots are 95% confidence intervals, calculated according to Loftus and Masson [20].

The error rates were generally low for Experiment 1 (see Table 1), and an analysis of variance (ANOVA) revealed no significant effects. Importantly, the error rates follow the same general pattern as the RT data (see Figure 1), indicating that the data are not likely contaminated by a speed-accuracy trade-off. The following analyses will only focus on the mean RT for the correct trials.

**Table 1:** Error rates for each condition in Experiment 1.

	Display size		
Condition	3	6	9
Target present: singleton	1.4	1.4	1.0
Target present: nonsingleton	4.0	6.8	8.6
Target absent	0.5	0.5	0.3

The results from Experiment 1 are plotted in Figure 1. A repeated-measures ANOVA on mean RTs for each subject with trial type (target singleton, target nonsingleton, or target absent) and display size (3, 6, or 9) as factors revealed a main effect for trial type, F(2, 26)  = 6.5, p<.01, and display size, F(2, 26)  = 26.4, p<.01, and a significant interaction of trial type by display size, F(4, 52)  = 3.0, p<.05. A brief inspection of the plot reveals that, on target present trials, participants responded more quickly when the target was the singleton (629 ms) than when a nontarget was a singleton (668 ms). An ANOVA with just the target present trial types as one factor (target singleton and nontarget singleton) and display size as the other factor supported this observation with a main effect of trial type, F(1, 13)  = 8.0, p<.05. There was also a main effect of display size, F(2, 26)  = 5.1, p<.05, however there was not a significant interaction between display size and trial type, F(2, 26)  = 1.7, p>.15. The slope relating response time to the number of elements was 3.5 ms/item for the target singleton trials, 2.7 ms/item for the target nonsingleton trials, and 9.5 ms/item for the absent trials. Search was very efficient, and each target present slope contains zero in its 95% confidence interval.

Even though participants were given the task of searching for a particular feature (right-tilted orientation), the participants were actually using singleton detection mode as revealed by the impact of the irrelevant feature of bar length. Note that participants responded more quickly on target absent trials (612 ms) than on target singleton (629 ms) or target nonsingleton (668 ms) trials. This suggests that the participants responded most quickly when all of the bars formed a homogeneous texture, and the uniquely-oriented target was absent, than when the target was present [11]. Thus when utilizing singleton detection mode, participants quickly and confidently responded when a singleton was not present, but took some additional time to respond that it was present. Note that previous researchers [7],[10] did not have target absent trials so there is little comparison for this aspect of the data. However this interpretation of the target-absent data is consistent with the conclusion that the participants were using the singleton detection mode strategy ([12] also report similarly-fast absent data). The RTs were particularly fast for the target absent and display size 3 trials, perhaps due to the greater variation in the location of the bars. Note that a smaller number of potential locations were allowed for smaller display sizes, however even the method used still results in relatively greater variation when fewer bars were present.

**Figure 2:**
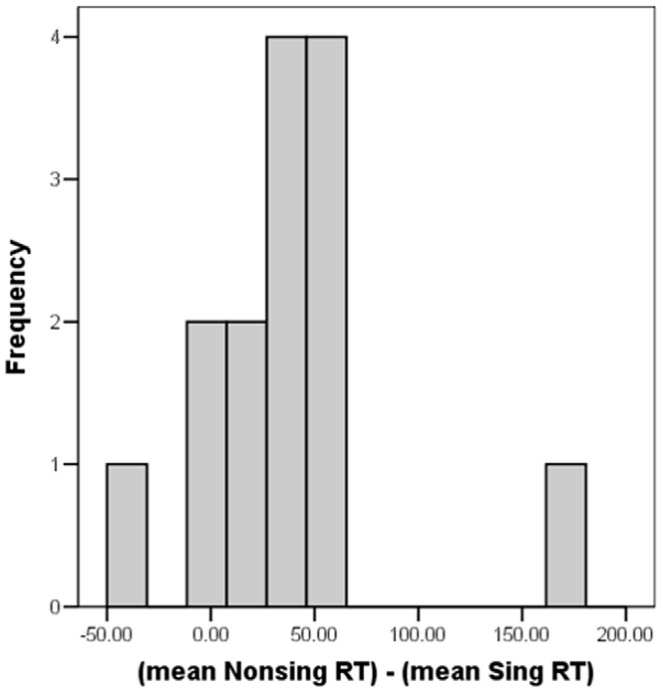
Histogram of the attentional capture effect for Experiment 1. The number of participants as a function of the difference between the mean RT of the nonsingleton target trials (Nonsing) and the singleton target trials (Sing) for each subject.

The mean difference between trials when the target was a singleton and when a distractor was a singleton was 39 ms. The distribution of the capture effect across individuals suggests that the irrelevant feature paradigm might be superior to the additional singleton paradigm when testing for attentional capture. An examination of the data for individual participants revealed that the average difference score ranged from −40 to 172 ms (see Figure 2). Only one subject might have even inhibited the large singleton, given the subject's large negative difference score (−40 ms). This subject also had RTs that averaged almost twice that of the other participants (target present mean RT 1109 ms versus the group mean RT 613 ms), suggesting that this one subject may have had some strategy that deviated from the other 13 participants. Overall there are individual differences in the magnitude of the distraction effect, however only this one subject's results were clearly in the opposite direction to that hypothesized. This implies that, for the most part, the participants are not engaging in some strategy to inhibit the output of the bottom-up process, as may have occurred in studies using the additional singleton method [13] in contrast to studies using the irrelevant feature paradigm [14].

### Experiment 2: Inefficient Search

**Figure 3:**
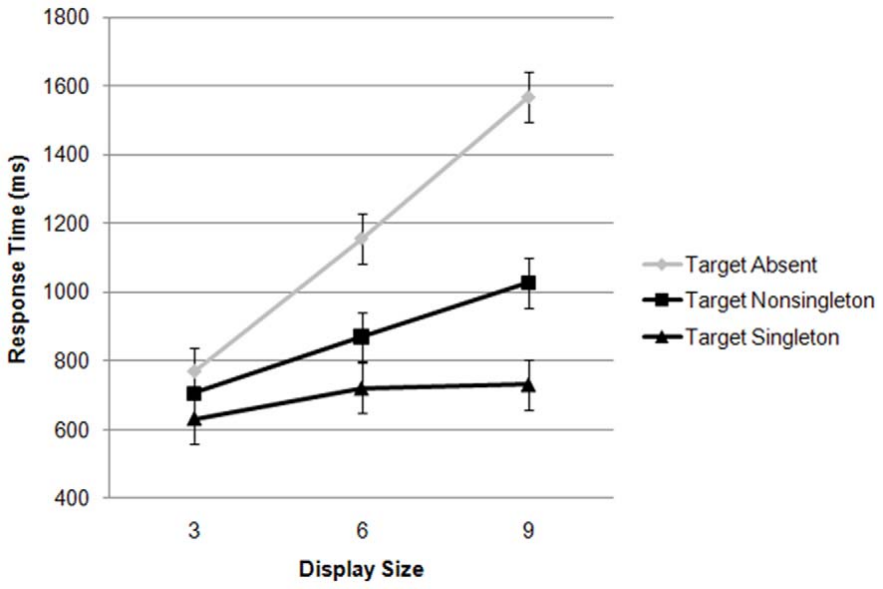
Experiment 2 results plotting response time as a function of display size for the target absent, target-present nonsingleton, and target-present singleton conditions.

The results from Experiment 2 are shown in Figure 3 and Table 2. The difference between the two target-present conditions (singleton, nonsingleton) was of primary interest, and these data were therefore subjected to a repeated-measures ANOVA of only the target present tirals. There were significant main effects of display size and target type, *F* (2, 14)  = 25.5, *p*<.001, and *F* (1, 7)  = 73.2, *p*<.001, respectively. The interaction of display size by target type was significant as well, *F* (2, 14)  = 35.8, *p*<.001.

**Table 2:** Error rates for each condition in Experiment 2.

	Display size		
Condition	3	6	9
Target present: singleton	0.0	0.8	1.3
Target present: nonsingleton	2.1	4.3	5.0
Target absent	0.0	0.0	0.1

The error rates are shown in Table 2. Importantly, the error rates follow the same general pattern as the RT data (see Figure 3), indicating that the interpretation of the data is not likely contaminated by a speed-accuracy trade-off.

The slope of the target singleton function was 17 ms per item versus 54 ms per item for the target nonsingleton function. The significant interaction of display size by target type suggests that the longer item was prioritized and captured attention. Importantly, in contrast to previous work using mixed trials [15],[16], it appears that blocking the trials did not results in less attentional priority in favor of the irrelevant large feature; in fact it resulted in somewhat shallower slopes than the previously reported mixed trials, and thus perhaps even greater attentional capture.

## Discussion

The results of both experiments have important contributions to the literature on attentional capture and visual search. First, Experiment 1 adds further evidence in support of a single-route hypothesis for visual search, in addition to the study by Zehetleitner et al. [6]. Note that their study did not find attentional capture when the irrelevant, additional singleton was present on 100% of the trials (consistent with [8]; and [17]. This experiment presents the first evidence that such capture can occur when the irrelevant feature appears with 100% frequency; the key difference here is that the large singleton could appear at the target location at chance, thus making it unlikely that the participants were induced to inhibit this feature in a goal-directed manner (as did the 50% frequency manipulation by Zehetleitner et al. [6]). Indeed this finding implies that the irrelevant feature paradigm, as used in the present study, might be superior to the additional singleton paradigm in that the likelihood of inhibiting the singleton is less.

Second, the results of Experiment 2 suggest that a large object can indeed capture attention in a stimulus-driven manner and independent of displaywide features of the task that might encourage a goal-directed bias for large items. It is concluded that these results are consistent with the stimulus-driven criteria published previously [3].

There is however another alternative to the stimulus-driven account of the results. The data are also consistent with a flexible, goal-directed mechanism of saliency detection. For example, in a study examining how target-nontarget similarity impacted attentional capture by an irrelevant bright feature, Proulx and Egeth [15] found that as salience (in terms of orientation contrast) became less useful for detecting the target, the participants relied less on salience for the task overall. Target-nontarget similarity was increased in the experiment (from 35 degrees to 15 degrees orientation contrast), and it was observed that an irrelevantly brighter feature captured attention less as the orientation contrast decreased. If relying on bottom-up feature contrast to detect the target can be flexibly applied, perhaps in a goal-directed manner, then perhaps attentional capture by a salient but irrelevant feature is modulated by the degree to which the attentional control settings of a participant take advantage of that contrast for target detection. This account would extend the ‘singleton detection mode’ of Bacon and Egeth [10] to inefficient search tasks, such as that used in Experiment 2 here, as well as provide an explanation for why attentional capture has been found in conjunction search as well [14].

It is important to note that this extension of singleton detection mode to inefficient search tasks emphasizes the point that the distinction between singleton search and feature search, laid out by Bacon and Egeth [10], cannot be distinguished by search slopes alone [18]. Furthermore, this account would modify the criteria set out by Burnham [3] by making the displaywide features of a task include the degree to which feature contrast (viz. salience) can be relied upon for target detection, independent of the efficiency of the task.

Future behavioral work will be necessary, in particular research examining whether attentional capture can take place in a purely stimulus-driven manner, while controlling the ability to use salience as an attentional control setting. Additionally, the feature of length has not been examined as extensively as luminance in the neurophysiological literature, however these results suggest that it would be a fruitful area of further study [19]. Certainly many singleton features might make a task easier by making an object easier to detect or discriminate [15], and determining whether attention is stimulus-driven or goal-directed is necessary to determine the neurophysiological mechanisms of attention [19].

## Materials and Methods

### Experiment 1

#### Participants

Naive participants (n = 14) all reporting normal or corrected-to-normal vision participated either in partial fulfillment of a course requirement or for payment after giving written informed consent. All experiments were conducted under the tenets of the Declaration of Helsinki and received Johns Hopkins University Institutional Review Board approval. These data were mentioned previously (but not with any details, analysis, nor figures) as pilot data in [14].

#### Apparatus and Stimuli

**Figure 4:**
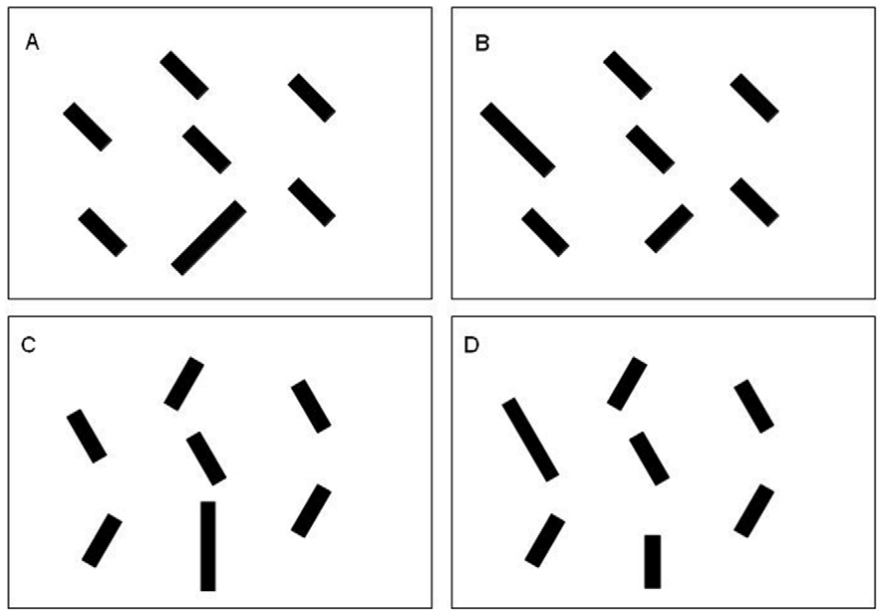
The figure depicts cartoons of the target present trial stimuli for Experiment 1 (panels A and B) and Experiment 2 (panels C and D). In panels A and B the target is a right-tilted bar (45 degrees from vertical) amongst left-tilted bars (−45 degrees from vertical). The target coincides with the irrelevant large feature in panel A; a nontarget coincides with the irrelevant large feature in panel B. In panels C and D the target is a vertical bar amongst bars tilted 30 degrees to the left and right of vertical. The target coincides with the irrelevant large feature in panel C; a nontarget coincides with the irrelevant large feature in panel D.

Participants were approximately 55 cm from the screen and a chin rest was used to stabilize their head location. The room was dimly lit such that the keyboard could be seen, but there was no glare on the monitor. Each stimulus display had a black background and three, six, or nine blue bars appeared for each trial. The nonsingleton bar size subtended 0.6 deg of visual angle in length and 0.15 deg in width. The large singleton bar subtended 0.9 deg in length and 0.15 deg in width. The target present trials are depicted in Figure 4.

The bars were dispersed in the cells of an invisible grid subtending 6 deg, 7 deg, or 8 deg of visual angle (with 7×7, 8×8, and 9×9 grid sizes, respectively) for a corresponding display size (3, 6, or 9 bars, respectively) to avoid increased crowding as display size increased. The bars were arranged within a subset of the cells of the grid, which were 1 deg apart, center-to-center; and the bar positions were each displaced by a random vertical and horizontal factor of+/− 0.2 deg to reduce rectilinear configuration effects (e.g., collinearity). The bar positions were selected randomly. The target bar was tilted to the right 45 deg (and appeared on half of the trials) and nontarget bars were tilted 45 deg to the left. There was no fixation point for any of the trials.

#### Procedure

Participants were instructed to look for “the bar rotated 45 degrees to the right among the bars tilted 45 degrees to the left.” A display of bars appeared on each trial and the subject pressed the “?/” key on a standard keyboard if the right-tilted target bar was present, and the “Z” key if it was absent. Participants were instructed to respond as rapidly as possible while making fewer than five per cent errors. They were informed of the probabilistic relationship between the singleton and the target. In particular, all participants were told that the length singleton was not predictive of the target and would only coincide with the target on 1/*d* of the trials, where *d* is the number of elements in the display. Incorrect responses were followed by a 1 kHz feedback tone for 100 ms and a recovery trial. Each trial began after a two-second inter-trial interval after each response was made. Each subject participated in 5 blocks of 108 trials per block. Each block of 108 trials included equal number of target absent and target present trials. There were also an equal number of trials for each display size. The number of each target-present trial type varied for each display size because the target was also the singleton on 1/d of the trials. All trial types were presented in a randomized order. At the end of each block, the participants received visual feedback including their reaction time and accuracy for that block. If their error rate exceeded five per cent, the participants were instructed to slow down and be more careful. Participants began with a practice block of 20 trials and each block began with three warm-up trials. Data from the practice, warm-up, incorrect, and recovery trials were not included in the RT analyses, however no RTs were trimmed.

### Experiment 2

#### Participants

Naive participants (n = 8) all reporting normal or corrected-to-normal vision participated either in partial fulfillment of a course requirement or for payment after giving written informed consent. All experiments were conducted under the tenets of the Declaration of Helsinki and received Queen Mary Research Ethics Committee approval.

#### Apparatus and Stimuli

The apparatus, stimuli, and procedure were based on Proulx and Egeth [15] and Yantis and Egeth [16]. Participants were 55 cm from the screen in a dimly lit room, and a chin rest was used to stabilize their head location. Three, six, or nine oriented bars appeared for each trial. The standard bar size subtended 0.6 deg of visual angle in length and 0.15 deg in width. The larger singleton bar was 50% longer, subtending 0.9 deg in length and 0.15 deg in width. The target present trials are depicted in Figure 4.

The bars were located randomly in the cells of an invisible grid subtending 6 deg, 7 deg, and 8 deg of visual angle for corresponding display sizes of 3, 6, and 9 bars. The bars were at least 1 deg apart, center-to-center; and the bar positions were displaced by +/- 0.2 deg. The target bar was vertical (and appeared on half of the trials) and nontarget bars were randomly tilted either −30 deg or +30 deg, with approximately half at each orientation.

#### Procedure

The display of bars appeared on each trial and the observer pressed one button if the vertical target bar was present, and another if it was absent. All observers were told that the singleton is not predictive of the target and will only coincide with the target 1/(display size) of the time, and thus would predominantly appear at nontarget locations. Incorrect responses were followed by a 1 kHz feedback tone for 100 ms and a recovery trial. Each trial began after a two second inter-trial interval after each response was made. At the end of each block, the participants received visual feedback including their reaction time and accuracy for that block. There were five blocks of 108 trials each for a total of 540 trials. Data from the practice, warm-up, incorrect, and recovery trials were not included in the RT analyses, however no RTs were trimmed.
